# Encapsulation of Probiotics within Double/Multiple Layer Beads/Carriers: A Concise Review

**DOI:** 10.3390/molecules29112431

**Published:** 2024-05-21

**Authors:** Sofia Agriopoulou, Slim Smaoui, Moufida Chaari, Theodoros Varzakas, Asli Can Karaca, Seid Mahdi Jafari

**Affiliations:** 1 Department of Food Science and Technology, University of the Peloponnese, Antikalamos, 24100 Kalamata, Greece; s.agriopoulou@uop.gr; 2 Laboratory of Microbial and Enzymatic Biotechnologies and Biomolecules, Center of Biotechnology of Sfax (CBS), University of Sfax, Road of Sidi Mansour Km 6, P.O. Box 1177, Sfax 3018, Tunisia; slim.smaoui@yahoo.fr (S.S.); moufida.chaari97@gmail.com (M.C.); 3 Department of Food Engineering, Faculty of Chemical and Metallurgical Engineering, Istanbul Technical University, 34469 Maslak, Turkey; cankaraca@itu.edu.tr; 4 Faculty of Food Science and Technology, Gorgan University of Agricultural Sciences and Natural Resources, Gorgan 49138-15739, Iran; 5 Halal Research Center of IRI, Iran Food and Drug Administration, Ministry of Health and Medical Education, Tehran 14158-45371, Iran

**Keywords:** probiotics, double/multiple layer coatings, encapsulation, functional food products

## Abstract

An increased demand for natural products nowadays most specifically probiotics (PROs) is evident since it comes in conjunction with beneficial health effects for consumers. In this regard, it is well known that encapsulation could positively affect the PROs’ viability throughout food manufacturing and long-term storage. This paper aims to analyze and review various double/multilayer strategies for encapsulation of PROs. Double-layer encapsulation of PROs by electrohydrodynamic atomization or electrospraying technology has been reported along with layer-by-layer assembly and water-in-oil-in-water (W_1_/O/W_2_) double emulsions to produce multilayer PROs-loaded carriers. Finally, their applications in food products are presented. The resistance and viability of loaded PROs to mechanical damage, during gastrointestinal transit and shelf life of these trapping systems, are also described. The PROs encapsulation in double- and multiple-layer coatings combined with other technologies can be examined to increase the opportunities for new functional products with amended functionalities opening a novel horizon in food technology.

## 1. Introduction

An increased food demand with specific health benefits arises from the adoption of healthier lifestyles. Consequently, the strategy for fruitful acceptance and marketing of new foods counts on (i) the idea of food quality and authenticity across the supply chain, and (ii) the boosted functionalities promoting added value [1,2,3]. Natural or processed foods fortified with bioactive natural compounds can be considered as functional food products [4,5,6,7]. Once managed in outlined qualitative and quantitative amounts, these new functional products could offer valuable health benefits to consumers [8]. In this vein, the expansion of probiotic (PRO) products is a significant research area for the market of functional foods [9]. Economic prognostications assume a rise to USD 7 billion for PRO dietary supplements between 2015 and 2025 worldwide [10].

Derived from the Greek word “for life”, PROs are defined as “live microorganisms that, when administered in adequate amounts, confer a health benefit to the host” [11]. Numerous bacterial species such as Lactobacillus, Bacillus Lactococcus, Streptococcus, Pediococcus, Bifidobacterium, and Propionibacterium are well recognized as PROs. Additionally, fungi and yeasts including Saccharomyces cerevisiae, S. Boulardii and S. carisbergensis, Aspergillus niger and A. oryzae are also investigated as PROs [12]. PROs are naturally considered as functional ingredients due to their wellness-enhancing capabilities [13]. In this line, PROs have various human health benefits such as the enhancement of intestinal microbial balance, prevention of pathogenic growth through the production of antimicrobial compounds, modulation and control of the innate immune systems, unveiling antimutagenic activities, and stopping/inhibiting cancers [14,15,16,17,18].

Currently, the most commonly utilized genera as PROs to support healthy intestinal function in humans are *Bifidobacterium* and *Lactobacillus*. Certain specific species within these groups have obtained the GRAS (Generally Recognized As Safe) status as conferred by the FDA (Food and Drug Administration) [19]. Other non-pathogenic microorganisms have also been used as PROs. Strains from *Pediococcus*, *Propionibacterium*, *Bacillus*, *Bacteroides*, *Streptococcus*, *Enterococcus*, *Escherichia*, and *Saccharomyces* are the most significant. Next-generation PROs with health benefits include *Akkermansia muciniphila*, *Faecalibacterium prausnitzii*, and *Eubacterium hallii* [20].

Practically, to apply therapeutic impacts on the host, the probable PRO strain viable cells in food products should be at least 10^6^ CFU/g (or CFU/mL) during the product’s shelf life [21]. However, the survival of a PRO is significantly affected by the rough gastrointestinal tract (GIT) conditions, as the low acidic pH of the bile acids and gastric environment [22]. In addition, it should be noted that numerous intrinsic and extrinsic factors have also been perceived as damaging the stability and viability of a PRO during food processing and preparation, along with the extended storage time [23,24]. To vanquish these contests, encapsulation methods have been realized to preserve the PRO’s viability. Encapsulation is an innovative approach in which PRO strains can be trapped inside a selective supportive membrane [25,26,27]. Once efficaciously applied, this technique could evade cell mass degradation, and accomplish a battered release in the gut in a satisfactory amount [28]. The result of this engineering process is an easy-to-handle encapsulated powder with uniform homogeneity through the food process [29].

The immobilization of cells through encapsulation techniques can be approximately considered as “macroencapsulation” and “microencapsulation” being affected by the size of the polymeric carriers [30]. Carriers developed through the macro-encapsulation process typically range from a few mm to cm while those in the range of 1–1000 μm are shaped through the microencapsulation process [30,31]. To protect PROs, several encapsulation technologies viz. emulsions, extrusion, coacervation, and drying methods such as freeze-drying, spray-drying, and fluidized bed drying have been implemented and industrialized [32]. In this regard, the proper encapsulation technique is identified based on the (i) characteristics of the PRO, (ii) the operative circumstances of the encapsulation process, (iii) the biomaterials’ feature, (iv) the suitable particle size for PRO loading without compromising the sensory quality of the end product, (v) the release mechanism/rate, and (vi) the storage conditions [33]. By preventing direct contact between the PRO and food ingredients, encapsulation techniques could retain the PRO’s viability throughout the food manufacturing process and long-term storage.

This article aims to analyze and review various double/multilayer strategies for encapsulation of PROs. In addition, here, we focus on the encapsulation of PROs within double/multilayer coatings and beads. Finally, their applications in food products are presented. Our manuscript provides relevant new insights and perspectives beyond the available reviews, and the key findings showed that the encapsulation of PROs in double- and multiple-layer coatings combined with other technologies was examined to increase the opportunities for new functional products. The comprehensive integration of these subtopics in a single review article is what makes this manuscript unique.

## 2. Encapsulation of Probiotics in Monolayer Beads/Carriers—Fundamentals and Mechanisms

Given the connection between human gut health and PROs, consumer interest in purchasing foods that contain PROs, or as supplements, is steadily increasing. At the same time, this increasing trend is also reflected in the global PROs market with a forecast referring to USD 911 million for 2026 and an annual growth rate of 8.3% [34,35]. The beneficial effects of PROs are correlated with both the strain used and the dose administered when consumed in an adequate population, and in all cases, these effects need to be proven both by in vitro experiments, in animals and human studies [36,37]. PRO microorganisms, to exert their potential valuable effects on the human body, must remain unaffected by harsh environmental conditions and maintain their characteristics when they colonize the human GIT [38].

In order to release the PROs from the encapsulation material, specific environmental conditions such as pH, temperature, and enzyme activity must be met [39]. Acidic gastric fluid contains water, hydrochloric acid, electrolytes, mucus, hormones, and digestive enzymes, e.g., lipase, renin, and pepsinogen. This particular composition, with a pH = 0.9 to 1.5–3.0, reduces the microbial population. In addition, the enzymes ribonuclease and deoxyribonuclease, as well as the pro-enzymes prochymotrypsin, protrypsin, proelastase, procarboxypeptidases, α-amylase, pancreatic lipase, and small intestine break down the PROs cells and are an important factor in their degradation [40]. Actually, the PROs strains, while they manage to reach GIT after oral administration through the gastric fluid, adhere only slightly to the intestinal mucosa, with the result that most of them are excreted with the feces [41].

**Figure 1:**
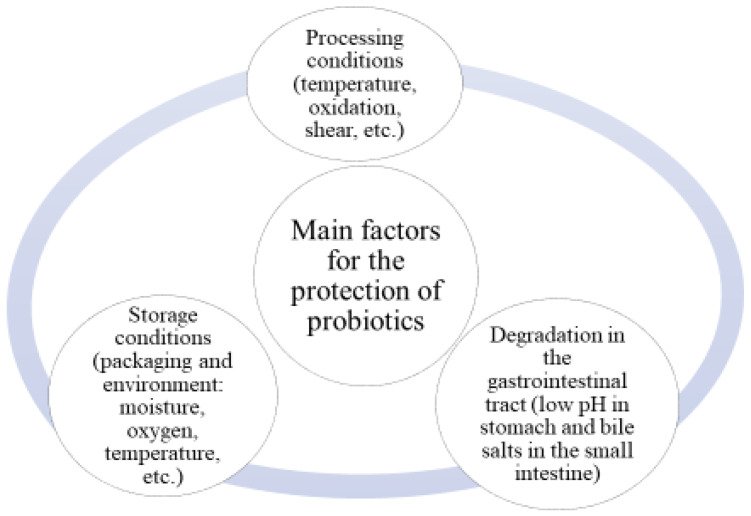
Main factors for the protection of probiotics (adapted from [45]).

In order to survive, distribute, and find their targets, PROs have to deal with a multitude of factors related to oxygen concentration, UV light, enzyme-mediated degradation, water activity, and antimicrobial action of bile salts, as well as competition phenomena caused by other bacteria [42]. Moreover, their survival in extremely low gastric pH remains one of the biggest challenges [43]. From the processing and production of food containing PROs to its consumption, the main concern is protecting the PROs cells (Figure 1). It is important to maintain the viability of probiotics through the conditions encountered during the manufacturing procedure (temperature, oxygen, shear, etc.), storage (moisture, oxygen, and temperature related to packaging and storage), and passage through the GIT (acidic pH of stomach and bile salts in the small intestine) [44].

Efforts currently being made by researchers are aimed at developing encapsulation technologies with the aim of protecting PROs in the human body and releasing them at targeted sites [46]. There are four main types of targeted delivery systems. The first one is called pH-responsive delivery systems and the pH of different sites of the body provides an idea for targeted delivery of PROs. Actually, the microenvironment in different sites of the human body has different pHs, and specific design is carried out according to the pH range of the target site. The second one is called the enzyme-specific response delivery system. In this case, the design depends on specific enzymes present in different microenvironments. The third one is called the immune-response delivery system, and usually targets specific sites that have a clear effect on PROs, such as lungs, tumors, etc. The last one is called targeted delivery to disease markers and signaling molecules. In disease states, inflammatory sites within the body produce far more disease markers and signaling molecules than normal. The presence of these disease markers and signaling molecules is being explored as a potential trigger mechanism for encapsulated PROs [46].

Through these techniques, significant viability rates of PROs are ensured as they approach the specific positions for which they are intended [47] while at the same time, their injuries or various cellular alterations are limited or reduced [38]. The wall that surrounds the PROs cells creating protective carriers during the encapsulation process is called the carrier [22] and aims to ensure the viability of the PROs throughout their journey from food to the colon [37], where they are most beneficial to the host [48]. Therefore, designing formulations and robust vehicles that will achieve targeted delivery remains as a challenge [49]. Wall, membrane, shell, external phase, or matrix material are some other words used for the carrier material [50].

The success of PROs encapsulation in terms of the functionality and viability of specific PRO strains is highly dependent on the encapsulation technique, the use of specific types of polymeric carriers, beads, carriers, matrices, and the type of microorganism. Among these widely used types are polysaccharides, lipids, and proteins or their chemically, physically, and enzymatically modified versions [47,51,52]. The use of polysaccharides and proteins helps to increase the durability of the structural integrity and cohesion while acting on the permeability of O_2_ or CO_2_ gases. On the contrary, the incorporation of lipids into the mixture enhances its resistance to water vapor [50]. By changing the composition of the coating and/or core material, or by modifying the chemical and/or physical treatments to which the carriers are exposed, it is possible to induce significant changes in the final characteristics of the carrier [50]. In the “microencapsulation” according to the size, carriers can be classified as macro- (>5000 µm), micro- (0.2 to 5000 µm), and nano-carriers (<0.2 µm) [50].

Since living cells of PRO strains must survive for an extended period and withstand the gastric environment and temperatures, various encapsulation techniques have been developed [42,53]. In encapsulation, a semipermeable membrane or matrix of a highly sensitive component is used [54]. The formation of a matrix during the process prevents the release of PROs into the product, making certain polymers more effective at encapsulation [55] and establishing an ideal microenvironment to support their survival and stability. Carriers are the ideal form of delivery of PROs to the GIT, particularly when addressing PRO formulations in liquid or powder form. Carriers can be present in solid form, with a soluble container that is either soft or hard [56]. The hard form is most commonly used in PROs and diluents, glidants, disintegrants or fillers are some of the excipients that carriers carry, the existence of which contributes to maintaining the physiology of PROs cells [57]. Different shell materials achieve the release and controlled delivery of the PROs cells from the carriers to selected sites or targets of the GIT [58].

**Figure 2:**
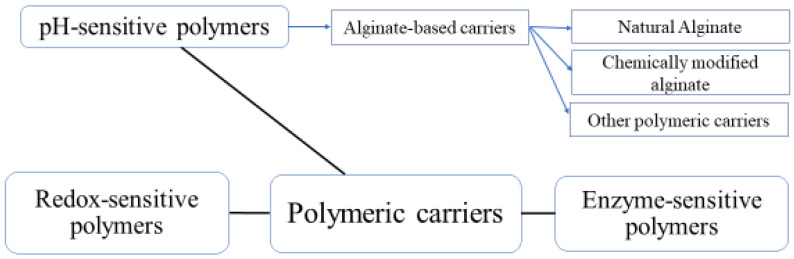
Polymeric carriers for enhanced delivery of probiotics (adapted from [44]).

Among the most commonly used proteins are whey proteins, caseins, and gelatin, while alginate, chitosan, and starch are the most known polysaccharides utilized as coating materials for PROs [47]. Fats of animal or plant origin, resins, and waxes are the main lipids used in encapsulation of PROs. Fish oil, butter, or lard can be used to produce fat of animal origin while sunflower oil, corn, or olive oil can be used to produce fat of vegetable origin [50]. Many researchers have documented the improved viability and high encapsulation efficiency of these coating materials for the encapsulation of PROs [59,60]. Depending on their functionality, the polymeric matrices used in encapsulation are distinguished into those related to sensitivity to pH, redox, and enzymes (Figure 2).

Polymers used in PROs encapsulation must be characterized by biocompatibility, biodegradability, processability, and PROs friendliness [44]. Other natural conventional biodegradable polymers used for the encapsulation of PROs are pectin, guar gum, dextran, chondroitin sulfate, cyclodextrin, xanthan gum, inulin, amylose, and locust bean gum shellac, while some synthetic polymers are Eudragit, polyvinyl acetate phthalate, hydroxypropyl ethylcellulose phthalate, cellulose acetate phthalate, and cellulose acetate trimellitate [44].

## 3. Monolayer versus Double/Multilayer Coatings

Spray-drying, freeze-drying (also referred to as lyophilization or cryodesiccation), spray chilling (also called congealing or spray cooling), electrospraying, extrusion, fluidized bed drying, layer-by-layer (LbL), and other physicochemical techniques such as coacervation and emulsification are some of the most commonly used techniques for encapsulation of PROs [38,50]. Among the commonly applied techniques in the industry for encapsulation of sensitive bioactive substances such as PROs is spray-drying, since it is extremely flexible in terms of operation with different wall materials, consumes relatively lower energy, and is characterized by high yield [59]. The core material is dispersed in a solution that includes the coating material, the resultant dispersion is homogenized and then sprayed into the drying chamber, which causes the solvent to evaporate in order to take a dry powder [47,59]. In freeze-drying, the PROs’ cell suspension is frozen at a low temperature, sublimated from ice to water vapor under vacuum conditions, and water is removed from the PROs solution to obtain a lyophilized powder [47]. In extrusion, a solution of polymer (typically a hydrocolloid) is mixed with the PROs cells. With the help of a syringe needle, the suspension is poured into a high-pressure solution of a cross-linking agent, resulting in the formation of a gel [61]. The emulsification process is defined by the dispersed phase including a cell polymer suspension and either vegetable oil, mineral oil, or an organic solution as the continuous phase. The emulsion results from the homogenization of the mixture and the surfactants.

During the creation of the carrier, its size is controlled, and this results in the approval of the product in terms of organoleptic characteristics [61]. In addition to that, size reduction may improve the application properties and physicochemical characteristics. The reduction in particle size also improves the consistency of the product and possible negative effects on its texture are eliminated [62]. PROs in the aqueous phase (W_1_) containing cryoprotectants such as disaccharides, proteins, polyalcohols, and complex mixtures are encapsulated through emulsions to improve the resilience of PROs against harsh GIT conditions [63].

Among the most extensively used polysaccharides as an encapsulation matrix is alginate. Alginate hydrogels (beads) could be an interesting option in PROs encapsulation. Their structure is composed of the two monosaccharides a-L-guluronic acid (G) and D-mannuronic acid (M). The fact that alginates do not dissolve in acidic gastric conditions makes them ideal for the protection of PROs in acidic gastric juice. In addition, their carboxyl groups form hydrogels with divalent cations [64]. In fact, the presence of divalent cross-linking cations creates a mild gelation, the result of which is the insolubilization of PROs formulations in acids. Among the various divalent cations (Mg^2+^, Sr^2+^, Ba^2+^), Ca^2+^ is widely used to form alginate hydrogels [64]. Alginic carriers (beads) are not only advantageous in enhancing the survival rate, stability, and targeted delivery of PROs but also present additional advantages related to simple, fast, and low-cost production. Alginates when combined with other biopolymers in hydrogel production are shown to be more effective in both encapsulation ability and viability of PROs compared to the use of alginates alone. Le and Trinh [65] managed to maintain the cell density of *Bacillus clausii*, *Saccharomyces boulardii*, and *L. acidophilus* until 120 min following double encapsulation (hydrogel of gelatin and alginate gels); also, their cell viability significantly improved.

There are many types of multilayer coatings, containing diverse materials that are typically effective for encapsulating PROs. Recently, Jeon et al. [51] achieved improved viability and storage stability of PRO bacteria under various temperatures after freeze-drying and enhanced their adhesion to intestinal cells, using quadruple-coated PRO strains containing red ginseng dietary fiber [51]. Coating materials included the combination of red ginseng dietary fiber (RDF) with basic amino acids (L-arginine, L-histidine, and L-lysine), tara gum, and rice protein powder. Sekhavatizadeh et al. [66] encapsulated *L. acidophilus* in sodium alginate and galbanum (*Ferula gummosa* Boiss) gum (second layer) microspheres to evaluate the survival under simulated GIT circumstances in PRO Tahini halva. Encapsulated *L. acidophilus* survived under refrigerated conditions for 18 days, the survival of viable cells improved up to 72 °C, while the survival rate under heat stress was 50.13%.

In terms of safety, ensuring the biocompatibility of encapsulation materials is paramount. Natural food-grade polymers like alginate and chitosan are GRAS, whereas synthetic polymers may require a more thorough safety evaluation. Meanwhile, both the encapsulation material and the PROs dosage need to meet the human daily intake limits [67].

High doses or frequent intake of PROs can increase the risk of intestinal flora imbalance [46].

The coating material can arrange in one, two, or more coating layers containing the core material. An ideal coating material should have the following desirable characteristics: be chemically inert with the core material; be able to seal and contain the core material inside the capsule; capability to provide protection against unfavorable conditions; and be sustainable and cheap. To date, no ideal coating exists yet that fits for all purposes, mainly because the coating characteristics cannot be simultaneously improved [50].

## 4. Different Multilayer Techniques for Encapsulation of Probiotics

PROs can be protected from harsh conditions by various double- and multiple-layer coatings as described below.

### 4.1. Electrohydrodynamic Atomization

Recently, electrohydrodynamic atomization (EHDA) or electrospraying technology has been utilized for downsizing carriers, as demonstrated by [68]. Electrohydrodynamic (EHD) processing is a method of generating liquid droplets through the application of a large electrical potential difference [68]. EHDA, characterized by a simple and adaptable experimental setup, is capable of generating monodisperse charged carriers from viscous polymeric solutions. This method offers several advantages over conventional encapsulation approaches such as freeze-drying and spray-drying. Charged carriers exhibit higher deposition efficiency in comparison to uncharged ones, and their movement can be readily controlled through external electric fields. This approach does not entail the use of severe temperatures or organic solvents and hence can be used for the encapsulation of live PROs cells [47,69]. The application of electrical force using an electrospinning technique allows for the formation of charged threads within micro/nano fibers from a polymer solution [70]. To date, electrospinning has been mainly used for the encapsulation of PROs in an electrospun monolayer using different biopolymers, although its use in multilayers is not limited. Recently, encapsulated *L. rhamnosus* GG (LGG) cells, in multilayer poly-lactic-co-glycolic acid-pullulan-poly-lactic-co-glycolic acid, electrospun nanofibers were reported; enhanced delivery of the cells and enhanced viability and shelf life after electrospinning was achieved [70].

Encapsulation of PROs using electrospinning is displayed in Table 1.

**Figure 3:**
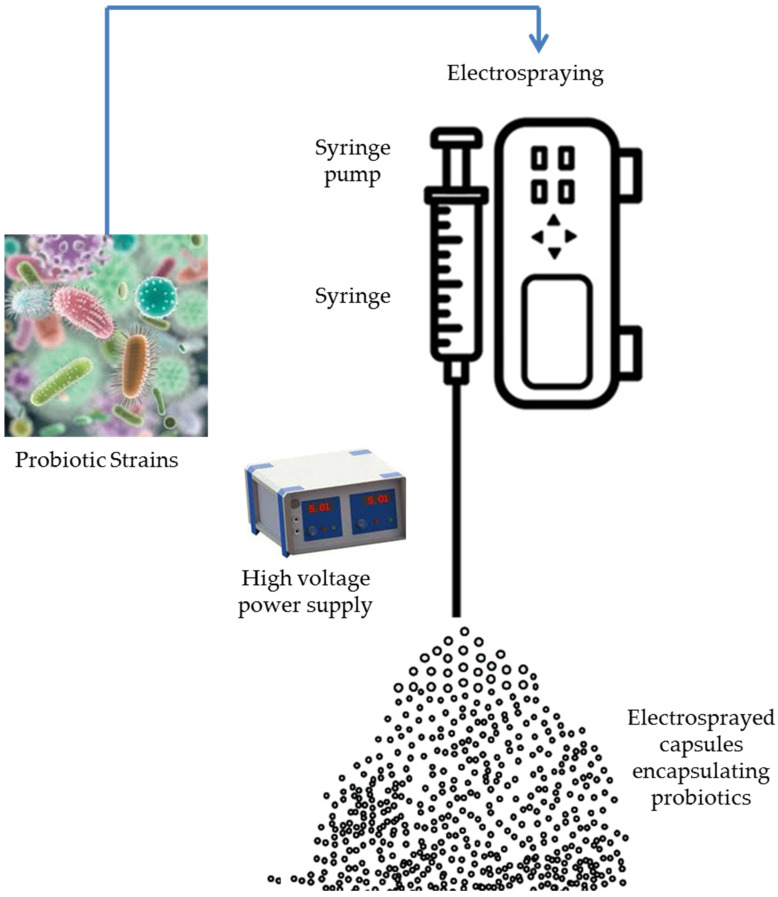
Electrospraying encapsulation of probiotics.

**Figure 4:**
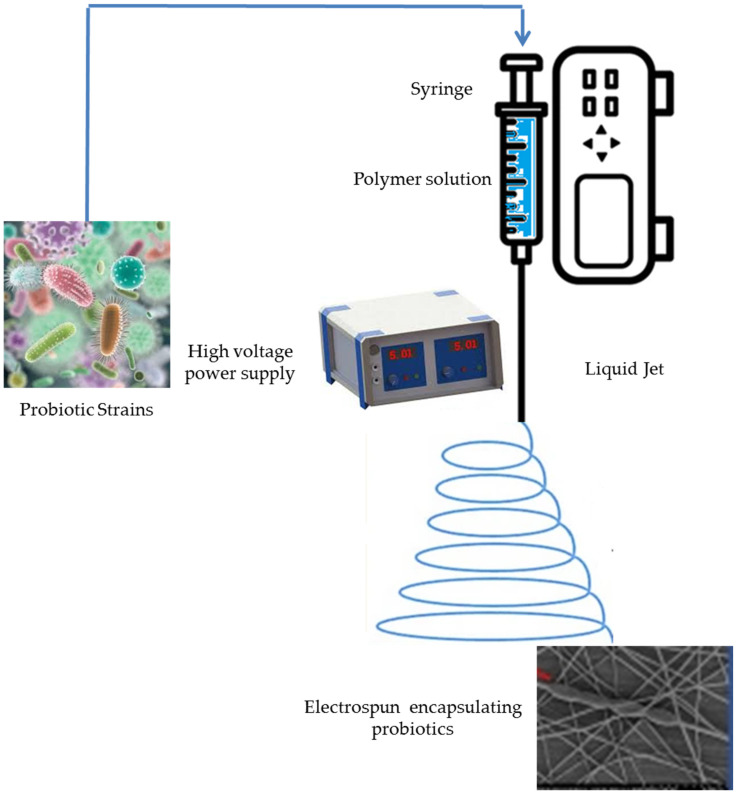
Electrospinning encapsulation of probiotics.

Electrohydrodynamic encapsulation employs similar physical methods as EHDA and can be considered as a version of EHDA [80]. The latter employed EHDA to co-encapsulate *Bifidobacterium lactis* and *L. plantarum* individually with either inulin or resistant starch within carriers made of Ca-alginate/chitosan. In this method, the extrusion of a polymeric solution including active materials is carried out through a capillary nozzle and atomization into ultra-fine droplets occurs due to powerful electrical forces. Solidification of the droplets into hydrogel particles following immersion in a gelling bath can occur [81]. The use of biodegradable and non-toxic wall materials or matrices, which serve to protect live cells, appears highly significant as well [82]. Electrohydrodynamic processes (EHD), including electrospraying and electrospinning, have recently emerged as innovative encapsulation approaches for PROs [83]. The surface of biopolymer solution droplets is being charged by high-voltage electrostatic fields, hence initiating the ejection of a liquid jet using a spinneret (Figure 3 and Figure 4, Table 1).

The electrical field corresponds to the reduction in the Taylor cone that then forms a steady and sustainable jet, which, due to its elongational viscosity, forms fibers. Approaching the electrode meter, the jet is narrower and forms an open spindle [84].

Modifying the dimensions and shapes of fibers and carriers generated through EHD is attainable by fine-tuning the EHD processing parameters such as applied potential, electric field, spinning distance, and flow rate, along with adjusting solution parameters including conductivity, viscosity, surface tension, and dielectric constant [85,86]. Different forms might appear in electrospraying, due to the Rayleigh–Plateau instability induced by surface tension. A jet breaks into droplets in the Taylor cone (a conical form). Electrical power allows the Taylor cone to distort the typical spherical meniscus shape [84]. In PROs encapsulation, jet mode and dripping mode appear as two modes of electrical atomization processes. Electrospinning facilitates the injection of LAB into solid delivery systems, concurrently achieving the dehydration of bacterial dispersion [87].

### 4.2. Layer-by-Layer Assembly

**Figure 5:**
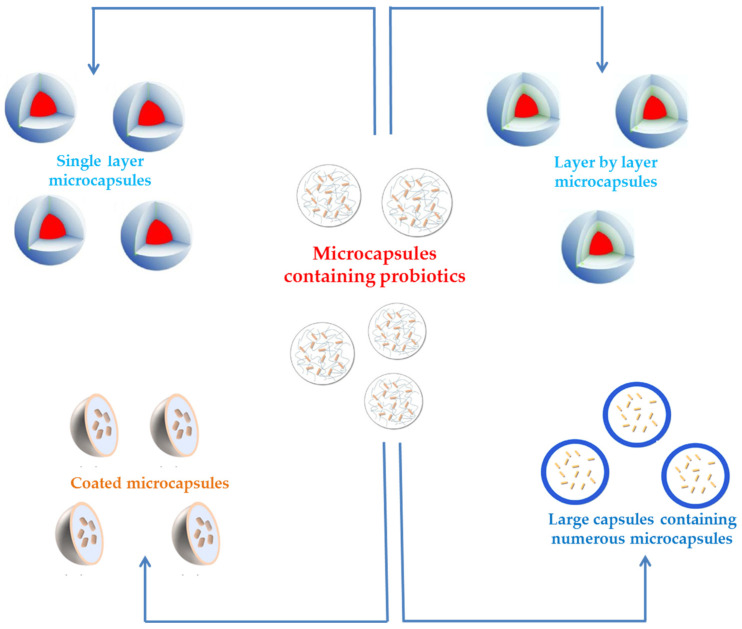
Layer-by-layer carriers for probiotics.

The LbL assembly has been employed for the fabrication of polymeric carriers endowed with diverse applications and release characteristics [88,89,90]. In the LbL technique, both natural and synthetic oppositely charged polymers are deposited in an alternating fashion onto a negatively charged mammalian cell membrane through electrostatic ad- sorption, thereby providing enhanced protection and a way to direct retention, proliferation, and growth on intestinal surfaces without requiring release from the encapsulating matrix [91]. The sequential adsorption of materials featuring opposing charges onto a template in a systematic manner, thus leading to the formation of a polyelectrolyte shell, is required for LbL assembly [92,93,94]. This technology presents an economical, readily available, and manageable approach for crafting multilayer carriers with adjustable digestive resistance, determined by factors such as the quantity, thickness, and barrier characteristics of the shell layers [88,92,93,95]. According to the literature, the preparation of resistant starch carriers with functional properties involves the construction of multiple calcium alginate layers around beads formed with calcium alginate and starch. It has also been indicated that enhancing the digestive resistance of starch in the interior of carriers and regulating its fermentation in the colon can be accomplished by creating multilayered sodium alginate shells around starch beads [96]. Figure 5 depicts layer-by-layer carriers for probiotics.

LbL deposition of soy β-conglycinin and high methoxylated pectin was achieved by preparation of fish oil-in-water emulsions using high shear mixing or homogenization at 500 or 3000 psi as reported by [97]. A carrier composed of anionic alginate and the LbL assembly [98] created cationic polycyclodextrins, with the target of inhibition and elimination of pathogenic bacteria. Similarly, triple-layer beads consisting of alginate, *Ferula assa-foetida* gum, and Zedo (*Amygdalus scoparia*) gum were used to encapsulate *L. reuteri* for application in a dairy dessert. Encapsulation was reported to enhance the viability of *L. reuteri* (7.5 log CFU/g) during storage [60]. Asgari et al. [44] produced multilayer PRO-loaded carriers. The LbL self-assembly process is a widely employed technique for PROs encapsulation (Table 2), relying on the consecutive adsorption of particles with opposing charges [99,100]. Layer-by-layer coating and multilayer carriers for probiotics are described in Table 2.

The LbL assembly of polyelectrolytes to create polyelectrolyte multilayer hollow carriers (PMCs) featuring a core–shell structure with various functional properties has become a well-established approach. PMCs exhibit diverse applications, including their potential use as delivery vehicles for controlled and targeted release [108].

### 4.3. Water-in-Oil-in-Water (W_1_/O/W_2_) Emulsions

**Figure 6:**
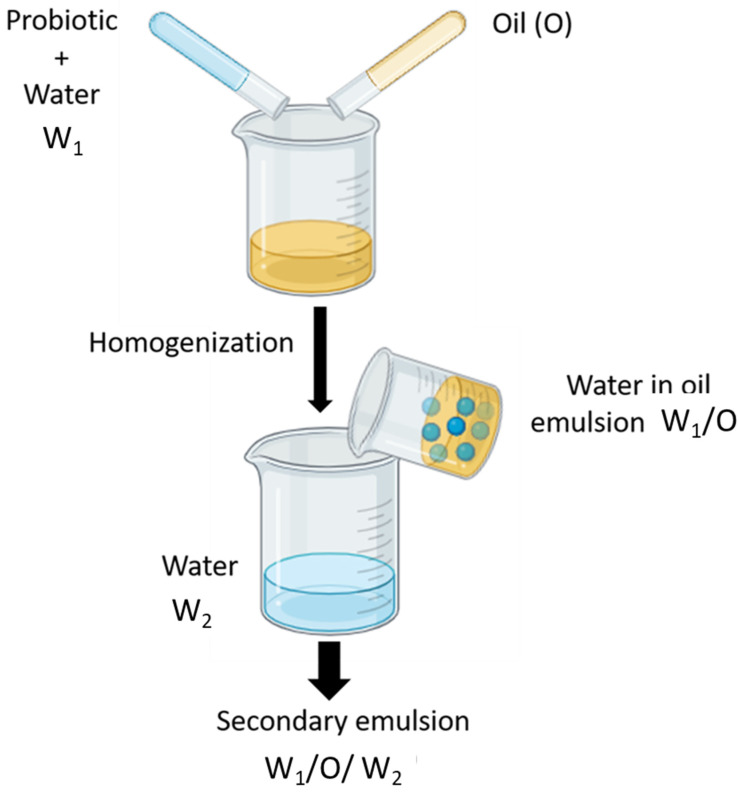
Water-in-oil-in-water (W_1_/O/W_2_) emulsions.

Emulsification of a W_1_/O emulsion in water results in a W_1_/O/W_2_ emulsion. This type of emulsion is denoted as “multiple emulsion” or “double emulsion.” Double emulsions can encapsulate PROs due to the ability to be integrated into the internal aqueous phase, protecting the external environment [109,110,111]. W_1_/O/W_2_ double emulsions are usually fabricated by a two-step process method. A water-in-oil (W_1_/O) emulsion is fabricated firstly, and then emulsified with external water phase [63]. Stability of a W_1_/O emulsion is the key step in preparation and this needs to be accomplished under high shear conditions. Following that, the re-dispersion of this emulsion takes place. This occurs in a hydrophilic emulsifier. High shear leads to the collision of water droplets and results in coalescence. Particle-stabilized Pickering emulsions (PEs) depend on Pickering stabilizers (Section 4.4) and constitute PROs encapsulation. Double emulsions established with PROs cells as the inner aqueous phase and various protective compounds have been reported by Ding et al. [112]. Figure 6 depicts water-in-oil-in-water (W_1_/O/W_2_) emulsions.

The interfaces of both W_1_/O and O/W_2_ were stabilized using soybean lecithin and polyglycerol polyricinoleate (PGPR), a widely used molecular surfactant [112]. Carboxymethyl konjac glucomannan–chitosan, a nano gel matrix, stabilized the outer aqueous phase. Eslami et al. [113] studied the formation and stabilization of multiple emulsions for *L. dellbrueckii* utilizing β-cyclodextrin (β-CD) inclusion complexes. A PRO-containing aqueous phase and oil phase with Span-80 constitute the initial emulsion (W_1_/O). This emulsion is then transferred to an outer aqueous solution of Tween-80 or β-CD and contains W_1_/O/W_2_ emulsion. Encapsulation of different PROs in double emulsions can be formulated with a variety of emulsifiers like sugar beet pectin, carboxymethyl konjac glucomannan-chitosan, PGPR, medium chain triglyceride (MCT) oil, Sweet whey, Inulin, and β-cyclodextrin (Table 3).

### 4.4. Multiple Pickering Emulsions

PEs have been employed in encapsulation, with differentiation in stabilization techniques, such as the use of hybrid or protein nanoparticles [120], along with multiple or high internal-phase PEs [121]. Pickering emulsions are stabilized by adsorbing nano- or micro-sized solid particles at the oil–water interface to prevent coalescence of the emulsion droplets. The large interfacial adsorption energies of Pickering emulsifiers allow for irreversible adsorption, unlike conventional emulsifiers [122]. PEs were prepared by stabilization of hydroxypropyl methylcellulose (HPMC), a representative anionic polymer, with chitosan and *Lactococcus lactis* IO-1 (*L. lactis IO-1*), as detailed by [123]. The PEs exhibited the health-promoting attributes of chitosan coupled with the bacteriocin produced by *L. lactis* exerting antibacterial activity. *L. lactis* negatively charged cells along with positively charged chitosan modified bacterial properties and formed the basis of a soft hydrophobic material for PEs. *L. plantarum* served as an emulsifier within PEs and their high internal phase following encapsulation with WPI/EGCG covalent conjugate nanoparticles. Hence, storage durability increased. A highly viscous or gel-like network is the characteristic of HIPEs achieved with a minimal oil fraction (φ) = 0.74 [124]. WPI-EGCG covalent conjugates forming nanoparticles were generated by a free-radical induction method [64]. This could lead to the stabilization of PEs. A double PE for loading *L. acidophilus* aiming at targeted delivery to the colon was developed by Wang et al. [125]. Double emulsions are considered highly advantageous in the triggered release and flavor masking.

LbL could generate multilayer emulsion self-assembly on double emulsion templates as designed carriers, thereby improving encapsulation and facilitating controlled release [126]. A typical formation of a multilayer emulsion involves the application of additional layers covering the emulsion droplets. Feng et al. [127] constructed this emulsion through LbL self-assembly, employing inversely charged biopolymers that interact through electrostatic attraction. Interfacial characteristics, e.g., size, charge, penetrability, and rheology can be regulated by sequentially depositing cationic and anionic biopolymers around emulsion particle templates [127]. Multilayer emulsions with thicker interface layers typically exhibit enhanced stability, resisting the coalescence and flocculation of emulsion droplets.

Ultrasound-assisted multilayer double PE carriers with WPI-EGCG covalent conjugates were reported to have a significant effect on the viability of *L. plantarum* strain liquid during pasteurization and GIT digestion [128]. The double emulsion produced under an ultrasonic intensity of 285 W exhibited a singular and narrow distribution, featuring the smallest droplet size. Subsequently, the double emulsion particles were coated with chitosan, alginate, and CaCl_2_. Chitosan and alginate are frequently employed as LbL materials due to their opposing charges. After pasteurization and GIT digestion, three to four coating layers exhibited comparable activity. However, formulations with three layers of coating were found to be the most effective for the encapsulation of *L. plantarum*.

HIPEs, also referred to as high-concentration emulsions, possess a droplet concentration surpassing the close packing limit, typically around 74% (*v*/*v*) according to Shi et al. [129]. At these elevated concentrations, the droplets tend to undergo deformation, adopting polyhedral shapes that are separated by thin films of the continuous phase. In comparison to HIPEs stabilized by conventional surfactants, HIP-PEs necessitate fewer stabilizers. They also exhibit higher internal-phase volumes, increased stability against coalescence, enhanced storage stability, and contribute to less environmental pollution [130]. Probiotic encapsulation using PEs is described in Table 4.

## 5. Applications of Multilayer Encapsulated Probiotics in Food Products

**Figure 7:**
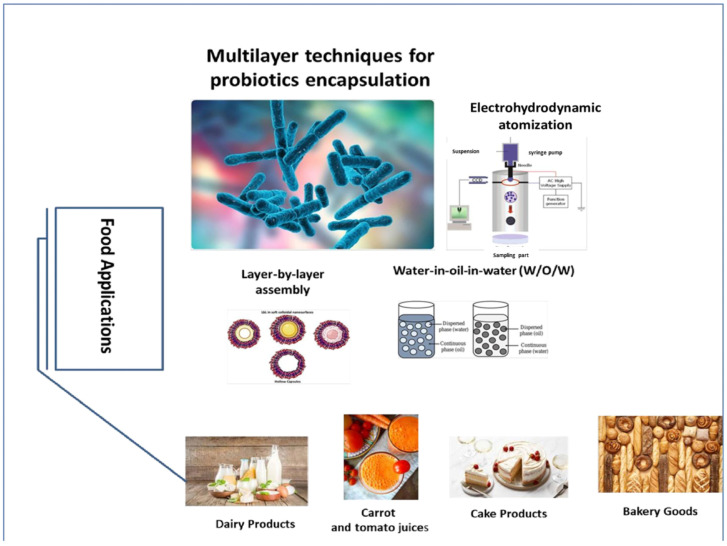
General scheme of multilayer techniques for probiotics encapsulation and their food application systems.

The utilization of encapsulated PROs in food systems has been extensively explored, finding common use in dairy products and more recently in nondairy alternatives (Figure 7). Pandey et al. [139] prepared double emulsion carriers enclosing the *L. plantarum* NCDC 414 and γ-aminobutyric acid (GABA). Under refrigeration at 4 °C, all carriers exhibited stability, with GABA encapsulation levels remaining >70% till 60 days. At 10^5^–10^7^ CFU/mL, the encapsulated LAB was viable and retained its entrapment even after exposure to sequential digestion. These authors concluded that ultrasonically produced PRO LAB carriers have the potential for targeted intestinal delivery and food formulations. He et al. [128] illustrated the influence of ultrasound-assisted multilayer W/O/W PE carriers on the viability of *L. plantarum* on pasteurization and gastrointestinal digestion. Coated with chitosan, alginate and CaCl_2_ at three to four layers possess comparable activity after LAB PRO pasteurization/GIT digestion. At five coating layers, multilayered carriers displayed the most viability; nonetheless, its particle size, measured at 108.65 μm, exceeded the limit of human oral sensory perception (80 μm). To produce PRO yogurt, Mahmoodi Pour et al. [140] established simple and multilayer emulsions by encapsulating *L. rhamnosus* and *L. plantarum*. Compared to free PROs, in which a notable loss of survival was observed, these authors stated that multilayer emulsion did not display a remarkable reduction in survival in yogurt. In addition, the encapsulation did not alter the organoleptic properties of the yogurt. Encapsulating PROs in simple emulsions led to a less homogenous structure in yogurt.

Jasińska et al. [141] prepared microbeads and microcapsules by extrusion as electrostatic and vibrating techniques. Compared to non-encapsulated strains, in the fermented nonmilk beverages, the *B. infantis* ATCC15697 immobilized in alginate or low-methoxyl pectin hydrogel particles meaningfully improved the survival rate of PROs strains during storage. Karimi et al. [60] described a dairy dessert containing *L*. *reuteri* ATCC 23272 encapsulated by sodium alginate, *Ferula assa-foetida* gum, and Zedo (*Amygdalus scoparia*) gums. Encapsulation enhanced the viability of the PRO strain at 7.5 Log CFU/g during storage. In addition, the PRO strain resistant to high temperatures (to 72 °C) contributed to the hardness value of the produced dessert. In addition, encapsulated *L. reuteri* pH value was closely stable throughout the storage period. In another study by Chen et al. [142], the impact of the xanthan–chitosan–xanthan system on *B. bifidum* BB01 viability in yogurt during 21 days of storage (at 25 and 4 °C) was investigated. Findings revealed that xanthan–chitosan–xanthan carriers and xanthan–chitosan carriers could enhance the survival of *Bifidobacterium* BB01 cells in yogurt. Core–shell capsules of *L. acidophilus* NCFM, prepared by alginate, locust bean gum, and mannitol, were effectively combined in mulberry tea [143]. In an acidic environment, the cells were well protected, and till the end of product storage (30 days) at 4 °C, the number of PRO LAB was 6.80 log CFU/mL, which encountered the minimum prerequisite for PROs (10^6^ CFU/mL).

PROs cultures including *L. plantarum*, *L. casei*, *L. fermentum*, *Sc. Boulardii*, and *Lysinibacillus sphaericus* were encapsulated by alginate-coated chitosan beads and introduced into carrot and tomato juices. The viable cell count of *Lysinibacillus sphaericus* increased from 6.5 to 8.9 log CFU/mL, and *Sc. boulardii* increased from 5.2 to 7.6 log CFU/mL between 24–42 h [144]. Over 5–6 weeks at 4 °C, the encapsulated cells showed higher viability compared to the free cells in tomato and carrot juices; nevertheless, the beads negatively affected the sensory properties of the produced juices. In another attempt, Nualkaekul et al. [102] investigated the impact of multilayer coating of alginate beads on the survival of encapsulated *L. plantarum* during storage in pomegranate juice at 4 °C at 6 weeks of storage; cell concentration in pomegranate juice was > 5.5 Log CFU/mL for double-coated beads. In contrast, for free cells and uncoated beads, the cells experienced mortality after 4 weeks of storage.

Arslan-Tontul et al. [145] incorporated double-layered carriers containing *Sc. boulardii*, *L. acidophilus*, and *B. bifidum* in three cake samples named cream-filled, marmalade, and chocolate-coated, after baking. For plain cake, carriers were inoculated into the center of the cake mix and baked at 200 °C for 20 min. These authors noted that double-layered carriers could enhance the survivability of PRO bacteria through the process of cake baking. In this line, cream-filled PRO cake samples demonstrated improved cell survivability during storage. During storage, cake staling had a partial impact on the sensorial features of the cakes and the cake samples remained consumable even after being stored for 90 days. To produce PRO bread, a fluidized bed-drying technique was applied by Mirzamani et al. [146] to encapsulate *L. Sporogenes*. Under baking conditions, double-layered carriers resulted in the highest heat resistance and, consequently, protected the coated PROs. By assessment of encapsulated PROs viability in bread, these authors depicted that the employment of chitosan and alginate in carriers could preserve *L. Sporogenes* and can be defined as a practical approach in PROs bread production. More recently, Sekhavatizadeh et al. [66] produced PRO halva by employing encapsulated *L. acidophilus* using sodium alginate and galbanum gum. Encapsulated *L. acidophilus* contained a viable count at an acceptable level (>10^6^ CFU/g) under refrigerated conditions for up to 18 days. In addition, during storage, the formed Tahini halva experienced a decrease in cell viability of 3.25 Log CFU/g.

Wong et al. [147] applied a dual coating to fresh-cut apple slices, initially using a bilayer of PRO *L. plantarum* 299v that was incorporated into an edible coating solution containing CMC, followed by a second zein coating. The apple slices were stored for 7 days at 4 °C, and throughout this period, *L. plantarum* 299v maintained stability at a level > 6 Log CFU/g. The bilayer PRO edible coating reduced weight loss, suppressed yeasts and mold growth, and an inhibition in the proliferation of spiked *Listeria monocytogenes* was observed during storage. Jantarathin et al. [148] demonstrated that encapsulation of *L. acidophilus* TISTR 1338 within a double-coated alginate bead with chitosan improved bacterial survival following freeze-drying. Moreover, the use of prebiotics including inulin and Jerusalem artichoke enhanced the viability of the encapsulated bacteria during the heating process. These authors concluded that this could illustrate the protection of PRO bacteria during the heating process in a shrimp-feeding machine.

There is evidence that PROs mixtures may be more effective than individual strains. For instance, a mixture of *Lb. acidophilus* W70, *Lb. casei*, *Lb. salivarius*, *Lactococcus lactis*, *B. Bifidum*, and *B. infantis*, as well as a mixture of *Lb. paracasei* B21060 and B21070 and *Lb. acidophilus* B21190 and a mixture of *Lb. casei* and *Lb. acidophilus* inhibit more successfully pathogen growth compared to each strain on its own. Different species may inhibit each other by producing antagonistic agents or by competing for either nutrients or binding sites within the GIT. Thus, a wide variety of genera in a multi-strain PROs mixture may decrease its effectiveness [25] Table 5 summarizes applications of multilayer encapsulated PROs in food products.

## 6. The Resistance and Viability of Probiotics Loaded in Multilayer Carriers

The aptitude of the wall materials to arrange a layer avoiding contact with severe conditions touches the survival of freeze-dried probiotics under GI conditions [149]. By exposure to simulated GI fluids, Moayyedi et al. [150] concluded that encapsulated *L. rhamnosus* with WPI/Persian gum/inulin displayed ~8 logs CFU/g. The buffering capacity of wall materials protects PROs against the GI, providing a good shield for probiotics [151,152]. Sometimes, the survival of probiotics is increased due to their acid and bile tolerance.

Sodium alginate microbeads crosslinked with calcium ions find limitations and cannot be stabilized in the stomach leading to rapid degradation [153]. The structure of microcapsules produced is preserved by complex coacervation under gastric conditions as reported by Barajas-Álvarez et al. [149]. In this study, the control release properties and viability of probiotics are regulated by the microcapsule composition. For instance, higher protection of *L. reuteri* is shown for gelatin: sodium caseinate compared to gelatin: GA.

The viability of PROs in foods could be touched by low pH, H_2_O_2_ and dissolved O_2_ content, presence of competing microorganisms and inhibitors, a_w_, and processing and storage T [154]. The resistance of sensitive PRO against adverse conditions can be augmented by the use of O_2_-impermeable containers, stress adaptation during cultivation, and the incorporation of micronutrients [155,156].

The practicality of the freeze-dried probiotic powders can be enhanced by the employment of a functional coating layer. Hot-melt coating includes the addition of coating material acting as a melt rather than a dispersion by a fluid bed coater [157,158], and minimization of exposure time to heat and moisture occurs. By hot-melt fluid bed coating, Jacobsen et al. [159] applied cetostearyl alcohol/olive oil/beeswax to *L. acidophilus* LA3 and *B. longum* BB536. Throughout intestinal transit, the coating system presented good release. Moussavi et al. [160] discussed the dependence of probiotic storage stability and gastrointestinal transit tolerance on species and carrier type. The addition of *Lacticaseibacillus rhamnosus* GG (LG), *Limosilactobacillus reuteri* ATCC 55730 (LR), *Bifidobacterium animalis* subsp. *lactis* BB-12 (Bb), *Propionibacterium jensenii* 702 (PJ), and combinations in orange juice and bottled water also affected them significantly.

Greater benefits to the consumer could be provided by probiotic combinations compared to single-strain preparations [161]. How well the cells in a probiotic product can survive in the GIT and then mediate the desired health benefit while passing through the human body is a question discussed thoroughly in the review by Wendel [162].

## 7. Conclusions

Recently, PROs have received increasing attention for their exceptional health benefits and biological potential. Nonetheless, the constrained stability observed during food processing and storage, especially under the harsh conditions of the GIT, significantly compromised the anticipated benefits, thereby limiting their applications. In this line, encapsulation of PROs within double/multiple layer coatings proposes an ample food solution. Once applied efficiently, the encapsulation technique has the potential to improve the PROs’ resistance to the harsh gastric environment and facilitate controlled release, ensuring effective delivery of PROs to the intended site of action. These novel delivery approaches for PROs are a humble, supple, and economical technology for the fabrication of various PROs multi-coating layers. On account of these structural benefits, the encapsulation of PROs in double- and multiple-layer coatings is revealed to (i) display high encapsulation efficiency, (ii) improve the bioavailability and stability, and (iii) accomplish targeted delivery and continued release. Recent progress in the encapsulation of PROs in double- and multiple-layer coatings was highlighted, along with their food potential applications. Presently, in the medical segment, the production of multilayer fiber structures at the industrial level is achievable; nevertheless, its employment in food science and agriculture is quiet in the initial phases of expansion.

The exploitation and changes of encapsulation of PROs in double- and multiple-layer coatings with other technologies can be examined to increase the opportunities for new products with amended functionalities. In this sense, partnerships between manufacturers and researchers are obligatory to construct industrial-level encapsulation of PROs in double- and multiple-layer coatings’ engines, hence enhancing throughput. Additionally, the regulation by the government agencies on the application of these new carriers in the food industry is highly desirable to guarantee the application of PROs food products. In the near future, the fruitful application of encapsulation of PROs in double- and multiple-layer coatings could open a novel horizon in food technology, presenting a commercialization opportunity.

## Figures and Tables

**Table 1:** Encapsulation of PROs using electrospinning.

Probiotic Strain	Polymer	Solvent	Processing Conditions	Average Diameter	Reference
*L. plantarum*	Ca-alginate (A)/chitosan (Ch)	A: water Ch: water at pH 3.5	9.5 kV 100 mm 5 mL/h	300–550 μm	[71]
*L. acidophilus*	Core: alginate/glycerol Shell: egg albumen and stearic acid	Water	8 kV 6 cm 10 mL/h	450 μm	[72]
*Bifidobacterium longum*	WPC, Fibersol^®^ (F) Maltodextrin (M), Zein (Z); PVP	F and M: water WPC: skimmed milk Z: ethanol PVP: water	Not specified	WPC: 2.47 μm F: 87 μm M: 1.95 μm	[73]
*B. animalis* subsp. *lactis* Bb12	Novel nanofiber mats consisting of chitosan (CS)/poly(vinyl alcohol) (PVA), inulin (INU) as a prebiotic	CS inacetic acid (0.5 M) PVA in water	18 kV Tip-to-collector distance: 15 cm 0.1 mL/h	117.5 to 217.6 nm	[74]
*Lactobacillus* strains	Gum Arabic (GA)-based nanofibers and pullulan	Deionized water	16 kV 0.4 mL/h, Tip-to-collector distance: 10 cm	Nanofibers with a smaller diameter	[75]
*L. plantarum*s	Polylactic acid and fructooligosaccharide	Dichloromethane and *N*, *N*-Dimethylformamide	16 kV, 0.1–0.25 mL/h	Electrospun fibers	[76]
	PVA/silk fibroin	n/a	n/a		[77]
*L. paracasei*	PVA and sodium-alginate	Water	15–27 kV 0.4–1.6 mL/h		[78]
	Eudragit L100 and Na-alginate	Alcohol	15 kV 1.0 mL/h		[79]

**Table 2:** Layer-by-layer coating and multilayer carriers for probiotics.

Matrix/Carrier	Food	Probiotic	Reference
Chitosan–alginate	-	*Bifidobacterium breve*	[101]
Chitosan-coated alginate beads	Pomegranate juice	*Lactobacillus plantarum*	[102]
Alginate–chitosan	Yogurt	*Lactobacillus acidophilus*	[103]
Chitosan/dextran sulfate multilayer polyelectrolytes	-	*Saccharomyces boulardii*	[104]
Nanostructured polyelectrolyte layers	-	*Lactobacillus acidophilus*	[105]
Chitosan and alginate	-	*Bacillus coagulans*	[106]
Single bilayer of alginate–chitosan and its double bilayer	-	*Lacticaseibacillus rhamnosus*	[107]
Chitosan and sulfated oat β-glucan	Oat β-glucan	*L. acidophilus*	[100]
Positively charged inner soy β-conglycinin and negatively charged outer high methoxyl pectin	Fish oil in water emulsions		[97]
Na-alginate shells around Ca-alginate/starch beads	Corn starch	*Bacteroides*, *Prevotellaceae*	[96]

**Table 3:** Probiotic encapsulation using W_1_/O/W_2_ emulsions.

Probiotic	Emulsifier	Reference
*L. salivarius NRRL B- 30514*	Sugar beet pectin	[114]
*L. reuteri*	Carboxymethyl konjac glucomannan–chitosan	[112]
*L. plantarum*	Polyglycerol poliricinoleate (PGPR)	[54]
*L. reuteri*	MCT oil (Miglyol^®^ 812)	[115]
*L. paracase*	PGPR	[116]
*L. rhamnosus*	Sweet whey	[117]
*L. rhamnosus*	Inulin	[118]
*L. delbrueckii*	β-cyclodextrin	[113]
*L. casei*	β-cyclodextrin	[119]

**Table 4:** Probiotic encapsulation using Pickering emulsions.

Emulsion	Probiotics	Pickering Stabilizer	References
O/W HIPE	*L. plantarum*	Whey protein isolate (WPI)/(-)-epigallocatechin-3-gallate	[63]
	*L. rhamnosus GG*	β-lactoglobulin-propylene glycol alginate composite nanoparticles	[111]
W/O	*None tested*	Butyl methacrylate derivatives	[131]
O/W	*L. casei*	Calcium alginate	[132]
W/W	*L. helveticus*	Microcrystal celluloses	[133]
	*L. helveticus* CICC 22536	Alginate	[134]
O/W	*L. rhamnosus* GG (LGG, ATCC 53103)	β-lactoglobulin-propylene glycol alginate	[111]
W/W	*L. plantarum*	Hydroxypropyl methylcellulose and dextran	[135]
O/W	*L. acidophilus* NRRL B-4495, *Lactiplantibacillus plantarum* NRRL B-4496	Gelatin	[136]
	*Lactobacillus acidophilus BC*	Nanoparticles	[125]
	*L. plantarum*	Alginate beads (emulbeads)	[137]
	*L. casei* ATCC 393	Silica particles	[138]

**Table 5:** Applications of multilayer encapsulated probiotics in food products.

Material	Encapsulation Technique	Purpose of the Encapsulation	Food Matrix	Encapsulated Probiotic Strain	Reference
Dextran γ-aminobutyric acid (GABA) and whey protein	Double emulsion (W1/O/W2) microcapsules	Stability, viability	Food formulations	*L. plantarum* NCDC 414	[139]
Chitosan(Chi), alginate (Alg), and CaCl_2_(Ca)	Ultrasound-assisted multilayer W/O/W PE	The role of ultrasonic homogenization on the morphology of W1/O/W2 double emulsions, viability	Granular food	*L. plantarum*	[128]
	Multilayer emulsions	Cell viability and physicochemical, rheological, structural, and sensorial properties of yogurts	Probiotic yogurts	*L. rhamnosus* and *L. plantarum*	[140]
Alginate or low-methoxyl pectin hydrogel particles	Extrusion	Survival rate	Fermented nonmilk beverages	*B. infantis* ATCC15697	[141]
Sodium alginate, *Ferula assa-foetida* gum, and Zedo (*Amygdalus scoparia*)	Microencapsulation	Physicochemical properties, sensory attributes, and probiotic survival	Dairy dessert	*L*. *reuteri* ATCC 23272	[60]
Xanthan–chitosan–xanthan	Double-layer encapsulation	Viability	Yogurt	*B. bifidum* BB01	[142]
Alginate, locust bean gum, and mannitol	Microencapsulation	Viability	Mulberry tea	*L. acidophilus* NCFM,	[143]
Alginate-coated chitosan beads	Microencapsulation	Viability	Carrot and tomato juices	*L. plantarum*, *L. casei*, *L. fermentum*, *Sc. Boulardii*, and *Lysinibacillus sphaericus*	[144]
Chitosan-coated alginate beads	Microencapsulation	Probiotic survival	Pomegranate juice	*L. plantarum*	[102]
Gum Arabic and β-cyclodextrin	Spray-drying and chilling	Textural and sensorial properties of the cake samples and probiotic survival	Cake	*Sc. boulardii*, *L. acidophilus* and *B. bifidum*	[145]
Alginate or xanthan gum as the first layer and gellan or chitosan as the outer layer	Microencapsulation	Viability	Bread	*L. Sporogenes*	[146]
Sodium alginate–galbanum (Ferula Gummosa Boiss) gum	Extrusion	Physicochemical, and textural properties of Tahini halva and probiotic viability	Tahini halva	*L. acidophilus*	[66]

## Data Availability

Not applicable.

## Abbreviations

EGCG epigallocatechin-3-gallate; EHD Electrohydrodynamic processes; EHDA electrohydrodynamic atomization; FDA Food and Drug Administration; GABA γ-aminobutyric acid; GIT gastrointestinal tract; GRAS Generally Recognized As Safe; HIPEs High internal phase emulsions; HPMC hydroxypropyl methylcellulose; LbL layer-by-layer; LGG *L. rhamnosus* GG; PGPR polyglycerol polyricinoleate; PMCs polyelectrolyte multilayer hollow carriers; PRO probiotic; RDF red ginseng dietary fiber; W_1_/O/W_2_ Water-in-oil-in-water; WPI whey protein isolate.
